# RNA Interference-Based Forest Protection Products (FPPs) Against Wood-Boring Coleopterans: Hope or Hype?

**DOI:** 10.3389/fpls.2021.733608

**Published:** 2021-09-10

**Authors:** Mallikarjuna Reddy Joga, Kanakachari Mogilicherla, Guy Smagghe, Amit Roy

**Affiliations:** ^1^Excellent Team for Mitigation, Faculty of Forestry and Wood Sciences, Czech University of Life Sciences Prague, Prague, Czechia; ^2^EVA.4 Unit, Faculty of Forestry and Wood Sciences, Czech University of Life Sciences Prague, Prague, Czechia; ^3^Laboratory of Agrozoology, Department of Plants and Crops, Faculty of Bioscience Engineering, Ghent University, Ghent, Belgium

**Keywords:** RNA interference, forest pests, double-stranded RNA delivery methods, enhancing RNAi efficiency, wood-boring coleopterans, symbiont mediated RNAi (SMR), forest protection products (FPPs)

## Abstract

Forest insects are emerging in large extension in response to ongoing climatic changes, penetrating geographic barriers, utilizing novel hosts, and influencing many hectares of conifer forests worldwide. Current management strategies have been unable to keep pace with forest insect population outbreaks, and therefore novel and aggressive management strategies are urgently required to manage forest insects. RNA interference (RNAi), a Noble Prize-winning discovery, is an emerging approach that can be used for forest protection. The RNAi pathway is triggered by dsRNA molecules, which, in turn, silences genes and disrupts protein function, ultimately causing the death of the targeted insect. RNAi is very effective against pest insects; however, its proficiency varies significantly among insect species, tissues, and genes. The coleopteran forest insects are susceptible to RNAi and can be the initial target, but we lack practical means of delivery, particularly in systems with long-lived, endophagous insects such as the Emerald ash borer, Asian longhorn beetles, and bark beetles. The widespread use of RNAi in forest pest management has major challenges, including its efficiency, target gene selection, dsRNA design, lack of reliable dsRNA delivery methods, non-target and off-target effects, and potential resistance development in wood-boring pest populations. This review focuses on recent innovations in RNAi delivery that can be deployed against forest pests, such as cationic liposome-assisted (lipids), nanoparticle-enabled (polymers or peptides), symbiont-mediated (fungi, bacteria, and viruses), and plant-mediated deliveries (trunk injection, root absorption). Our findings guide future risk analysis of dsRNA-based forest protection products (FPPs) and risk assessment frameworks incorporating sequence complementarity-based analysis for off-target predictions. This review also points out barriers to further developing RNAi for forest pest management and suggests future directions of research that will build the future use of RNAi against wood-boring coleopterans.

## Graphical Abstract

Three-tier impact of forest protection products (FPPs).

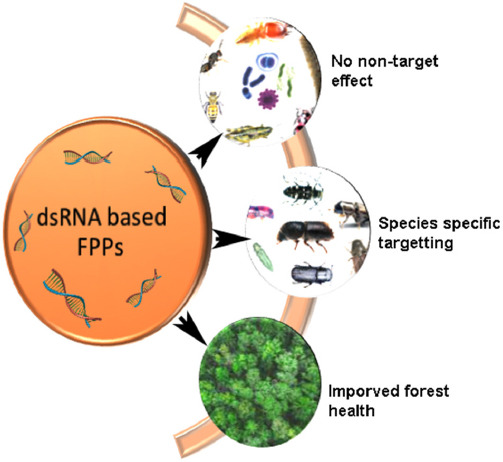

## Introduction

Forests are of immense importance due to their socio-economic and ecosystem services (Pan et al., 2013). However, a decline in conifer forests is ongoing worldwide at an unprecedented rate due to a rise in temperature, drought stress, windthrows, and pest infestation (Safranyik et al., 2010; Hlásny et al., 2019). Most of the insect pest population outbreaks are temperature-dependent, and climate-driven intensification in the frequency, severity and cyclicity of forest pest outbreaks is already well documented (Bentz et al., 2010, 2019; Cudmore et al., 2010; de la Giroday et al., 2012; Haynes et al., 2014; Bentz and Jönsson, 2015). Besides, forest insects have expanded their geographic range by exploiting native hosts previously unexplored due to low temperature (Williams and Liebhold, 2002; Cipollini and Rigsby, 2015; Ramsfield et al., 2016). Such range expansion also causes widespread tree mortality, decreasing forest productivity and carbon storage and substantially enhancing discharge from the decay of dead tree woods (Kurz et al., 2008). Thus, severe depletion of trees due to pest outbreaks or range expansion may cause trajectories outside the resilience limits of forest ecosystems resulting in irreversible ecosystem regime shifts (Dhar et al., 2016).

Coleopteran forest pests such as Emerald ash borer (*Agrilus planipennis*, EAB), Asian longhorn beetles (*Anoplophora glabripennis*, ALB) and bark beetles took advantage of ongoing climate change and cause severe damage to the forests worldwide (Aukema et al., 2011; Meng et al., 2015; Hlásny et al., 2019). For instance, EAB is a devastating, tree-killing phloem-feeding beetle from northeastern Asia recently invaded North America through solid wood packaging material (Poland and McCullough, 2006). EAB has already killed millions of North American Ash (*Fraxinus* sp.) and became one of the costliest insect pest invaders in American history (Aukema et al., 2011). EAB larvae disrupt the translocation of essential nutrients and water in the infested plants while feeding on phloem tissues, leading to the death of the Ash trees within 3–4 years of infestation (Haack et al., 2002). Management of these notorious tree killers is a daunting task, and superior methods can bring hope. ALB, similar to EAB, is native to China and Korea and is a globally recognized invader with a history of attacking more than 100 different species of trees (Haack et al., 2010; Meng et al., 2015). Estimation of loss due to ALB infestation would be a staggering $889 billion if the ALB population left uncontrolled (McKenna et al., 2016). Interestingly, trunk injection of systemic insecticides such as imidacloprid was documented effective against ALB infestation. However, the cost and environmental impact of deploying chemical pesticides jeopardize such strategies and call for better alternatives for ALB management.

Bark beetles (Coleoptera: Curculionidae: Scolytinae) are the most severe and destructive pests of conifer forests worldwide (Fairweather, 2006; Keeling et al., 2016; Hlásny et al., 2019). Since abiotic factors are primary drivers for bark beetle population growth (Biedermann et al., 2019), outbreaks of these aggressive forest pests are expected to increase frequency and severity due to ongoing climate change (Kurz et al., 2008). Warming temperatures promote bark beetle population growth due to reduced winter mortality and development time by allowing additional generations per year. A recent study featuring a tree-ring iso-demographic approach further supports the notion that temperature is more critical than drought for amplifying the eruptive bark beetle outbreaks (Pettit et al., 2020). Most bark beetle species breed on weak and dead trees during an endemic stage serving a crucial function in the forest ecosystem by recycling the nutrients from the dead plant tissues. However, once the bark beetle population increases to an epidemic level, they start attacking the healthy trees leading to an outbreak (Fairweather, 2006; Boone et al., 2011; Hlásny et al., 2019). Currently, frequent outbreaks have been a major disturbing factor for conifer forests in Europe and North America (Bentz et al., 2010; Meddens et al., 2012; Hicke et al., 2016; Hlásny et al., 2019; Lubojacký, 2019) that affects forest ecosystem functioning (Grégoire et al., 2015; Thom and Seidl, 2016), climate and carbon loss mitigation, water retention (Grégoire et al., 2015) and country economy via losses in timber and tourism revenue (Holmes, 1991; SFA, 2010; Pye et al., 2011; Arnberger et al., 2018; Cahyanto et al., 2018). Some aggressive bark beetles, such as the southern pine beetle (*Dendroctonus frontalis*) and the mountain pine beetle (*D. ponderosae*), undergo a substantial range expansion in the US due to favorable warmer climate and cause frequent outbreaks leading to catastrophic tree loss (Regniere, 2003; Chen and Goodwin, 2011; Lesk et al., 2017).

Several conventional approaches such as sanitation felling (Wermelinger, 2004; Seidl et al., 2016), removal of wind felled trees (Leverkus et al., 2018), and deployment of pheromone-baited and poisoned log tripod traps (Wermelinger, 2004) is used for the last few decades to manage the bark beetle population levels in endemic phase. However, the success of all these approaches is questionable in managing the recent bark beetle outbreaks (Billings, 2011; Hlásny et al., 2019). Furthermore, similar to other wood-boring forest insects, several synthetic pesticides have also been used to suppress bark beetles over the past years (Williamson and Vité, 1971). However, many of these compounds caused other problems such as environmental pollution, detrimental effects on non-target organisms, and widespread pesticide resistance (Feder, 1979; Baum et al., 2007; Billings, 2011). Therefore, questions have been raised about the feasibility, effectiveness, and purpose of conventional phytosanitary measures. Hence, novel and aggressive management of these devastating coleopteran wood-boring forest pests is the highest priority in the Anthropocene.

**FIGURE 1:**
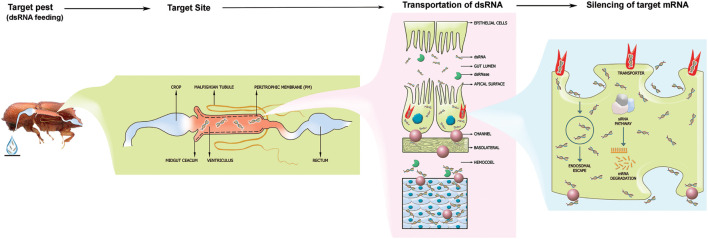
The fundamental mechanism of RNAi from a coleopteran pest control standpoint. The target pest is feeding on the species-specific, gene-specific dsRNA droplet. After ingestion, the dsRNA will reach the midgut and passes through the peritrophic membrane. Subsequently, the dsRNA will be up-taken by the gut epithelial cells through the scavenger receptor-mediated endocytosis pathway and the transmembrane Sid-1 channel protein-mediated pathway. Further, the dsRNA is exported to hemocoel and fat bodies connected all over the body. The siRNA mechanism will be activated and triggers gene silencing upon dsRNA uptake by cells. Endosomal escape of the silencing signal resulting in RNAi efficiency within coleopteran pest induces RNAi effect.

RNA interference (RNAi) is an evolutionarily conserved post-transcriptional gene silencing mechanism, which is triggered by exogenous double-stranded RNA (dsRNA) (Fire et al., 1998; Zhu and Palli, 2020). Thus, RNAi becomes a promising tool for forest pest management in this era of genomics (Baum et al., 2007). Recent advancements in sequencing technology and platforms lead to higher availability of coleopteran forest pest genomes and transcriptomes that can serve as valuable resources for species-specific dsRNA design (Keeling et al., 2012, 2013; Scully et al., 2013; McKenna et al., 2016; Powell et al., 2020). It was pretty well known that coleopteran insects are usually susceptible to RNAi (Figure 1; Baum et al., 2007; Zhu et al., 2011; Palli, 2014; Prentice et al., 2015; Ulrich et al., 2015; Fishilevich et al., 2016; Li et al., 2018a; Bramlett et al., 2020; Mehlhorn et al., 2021; Willow et al., 2021). Recently, Yoon et al. (2018) reported the underlying cause of coleopteran insect susceptibility toward RNAi. With intriguing evidence of RNAi susceptibility in coleopterans, researchers started exploring the potential of RNAi in managing coleopteran forest pests (Table 1). Recent high-quality publications demonstrated the entomotoxicity of RNAi against wood-boring coleopteran forest pests such as southern pine beetle, mountain pine beetles, emerald ash borer, Asian longhorn beetles, and Chinese White pine beetle (Rodrigues et al., 2017a, b; Rodrigues et al., 2018; Kyre et al., 2019; Dhandapani et al., 2020a, b; Kyre et al., 2020). However, the potential of RNAi in coleopteran forest pest management is not yet comprehensively summarized elsewhere. Hence, it is essential to capture all aspects of such studies together and critically evaluate the future potential of RNAi against wood-boring coleopteran pest management. The current review focuses on synthesizing key challenges for RNAi-mediated forest pest management (Table 2). It can also serve as a valuable source of information for general foresters, private forest owners, forest managers, and researchers worldwide who are currently using RNAi or planning to use RNAi as a tool against coleopteran pests inside the forests.

**TABLE 1:** List of RNAi experiments for wood-boring coleopteran pests.

S. No.	Author	Test organism	Common name	Target gene	Accession no.	Molecule (dsRNA/siRNA)	Size (bp)	Life stage tested	Mode of delivery	Concentration (μg/μL)	Measurement endpoints
1	Rodrigues et al., 2017b	*Agrilus planipennis*	Emerald ash borer	COP	Not informed	dsRNA	247	Larvae	Feeding	3 μg	24% mortality
				IAP	Not informed	dsRNA	272	Larvae	Feeding	3 μg	33% mortality
				IAP		dsRNA	272	Larvae	Feeding	10 μg	78% mortality
				IAP		dsRNA	272	Larvae	Feeding	1 μg	30% mortality
				IAP		dsRNA	272	Larvae	Feeding	6 μg	35% mortality
2	Rodrigues et al., 2018	*Agrilus planipennis*	Emerald ash borer	HSP	XM_018474521.1	dsRNA	468	Larvae	Feeding	10 μg	90% mortality
				HSP		dsRNA	468	Larvae	Feeding	1 μg	67% mortality
				HSP		dsRNA	468	Adults	Feeding	10 μg	40% mortality
				Shi	XM_018465318.1	dsRNA	483	Larvae	Feeding	10 μg	90% mortality
				Shi		dsRNA	483	Larvae	Feeding	1 μg	∼40% mortality
				Shi		dsRNA	483	Adults	Feeding	10 μg	30% mortality
				Shi + HSP	XM_018465318.1 + XM_018474521.1	dsRNA	483 + 468	Adults	Feeding	1 μg (500 ng/ μl each)	90% mortality
3	Zhao et al., 2015	*Agrilus planipennis*	Emerald ash borer	AplaScrB-2	KJ634683	dsRNA	475	Adults	Injection	200 ng	The expression of AplaScrB-2 was 73% repressed on day 3 and 90% repressed on day 6
4	Leelesh and Rieske, 2020	*Agrilus planipennis*	Emerald ash borer	Shi	XM_018465318.1	dsRNA	483	Larvae	Feeding (recombinant bacteria expressing dsRNA)	3 μL of bacterial suspension	69.44% mortality
				HSP	XM_018474521.1	dsRNA	468	Larvae			46.66% mortality
5	Dhandapani et al., 2020a	*Anoplophora glabripennis*	Asian longhorned beetle	IAP	XM_018711271.2	dsRNA	386	Larvae	Injection	10 μg	100% mortality
				Prosβ5	XM_018713596.1	dsRNA	377	Larvae	Injection	10 μg	80% mortality
				RpL6	XM_018709657.1	dsRNA	433	Larvae	Injection	10 μg	60% mortality
				Cas	XM_018707893.1	dsRNA	429	Larvae	Injection	10 μg	60% mortality
				Surf4	XM_018716988	dsRNA	357	Larvae	Injection	10 μg	60% mortality
				Ebony	XM_018713129	dsRNA	413	Larvae	Injection	10 μg	60% mortality
				Actin	XM_018721905.1	dsRNA	436	Larvae	Injection	10 μg	50% mortality
				SNF7	XM_018722997.1	dsRNA	342	Larvae	Injection	10 μg	50% mortality
				Dre4	XM_018708786.1	dsRNA	314	Larvae	Injection	10 μg	50% mortality
				Prosα6	XM_018714266.1	dsRNA	374	Larvae	Injection	10 μg	50% mortality
				Sec61α	XM_018707923.1	dsRNA	443	Larvae	Injection	10 μg	50% mortality
				VhaSFD	XM_018721020.1	dsRNA	410	Larvae	Injection	10 μg	50% mortality
				Unc-104	XM_018711981.1	dsRNA	406	Larvae	Injection	10 μg	40% mortality
				Rpn11	XM_018719490.1	dsRNA	441	Larvae	Injection	10 μg	40% mortality
				Sam-S	XM_018717099.1	dsRNA	425	Larvae	Injection	10 μg	40% mortality
				SSK	XM_018724815.1	dsRNA	326	Larvae	Injection	10 μg	40% mortality
				MESH	XM_018707459.2	dsRNA	341	Larvae	Injection	10 μg	40% mortality
				GW	XM_023456527.1	dsRNA	482	Larvae	Injection	10 μg	40% mortality
				IAP	XM_018711271.2	dsRNA	386	Adults	Injection	10 μg	100% mortality
				SNF7	XM_018722997.1	dsRNA	342	Adults	Injection	10 μg	100% mortality
				Shi	XM_018714700.1	dsRNA	427	Adults	Injection	10 μg	100% mortality
				Dre4	XM_018708786.1	dsRNA	314	Adults	Injection	10 μg	100% mortality
				Prosβ5	XM_018713596.1	dsRNA	377	Adults	Injection	10 μg	100% mortality
				Sec61α	XM_018707923.1	dsRNA	443	Adults	Injection	10 μg	100% mortality
				Sar1	XM_018718347.1	dsRNA	421	Adults	Injection	10 μg	∼80% mortality
				SSK	XM_018724815.1	dsRNA	326	Adults	Injection	10 μg	∼80% mortality
				Prosα6	XM_018714266.1	dsRNA	374	Adults	Injection	10 μg	∼80% mortality
				IAP	XM_018711271.2	dsRNA	386	Larvae and adults	Feeding	12 μg	No significant mortality in larvae and adults were observed and also did not notice knockdown of the IAP gene after feeding dsRNA
6	Dhandapani et al., 2020a, b	*Anoplophora glabripennis*	Asian longhorned beetle	IAP	XM_018711271.2	dsRNA	386	Larvae	Feeding	2 μg, 5 μg, 10 μg/day for 3 days	17%, 67% and 90% mortality
				SNF7	XM_018722997.1	dsRNA	342	Larvae	Feeding	2 μg, 5 μg, 10 μg/day for 3 days	25%, 50% and 75% mortality
				SSK	XM_018724815.1	dsRNA	326	Larvae	Feeding	2 μg, 5 μg, 10 μg/day for 3 days	17%, 67% and 80% mortality
7	Kyre et al., 2019	*Dendroctonus frontalis*	Southern pine beetle	HSP	XM_019906798.1	dsRNA	315	Adults	Feeding	10 μg	100% mortality
				Shi	XM_019900326.1	dsRNA	342	Adults	Feeding	10 μg	86.67% mortality
				IAP	XM_019910372.1	dsRNA	341	Adults	Feeding	10 μg	20% mortality
8	Kyre et al., 2020	*Dendroctonus ponderosae*	Mountain pine beetle	HSP	Not informed	dsRNA	351	Adults	Feeding	2.5 μg	∼85% mortality
				Shi	Not informed	dsRNA	379	Adults	Feeding	2.5 μg	∼80% mortality
				IAP	Not informed	dsRNA	370	Adults	Feeding	2.5 μg	∼75% mortality
9	Li et al., 2018b	*Dendroctonus armandi*	Chinese white pine beetle	CSP2	AGI05172.1	dsRNA	Not informed	Adults	Injection	200 ng	Antennal EAG activity reduced in response to host volatiles [(+)-α-pinene, (+)-β-pinene, (−)-β-pinene, (+)-camphene, (+)-3-carene, and myrcene]
10	Fu et al., 2019	*Dendroctonus armandi*	Chinese white pine beetle	DaAqp12L	XP_018562473.1	dsRNA	Not informed	Larvae	Injection	200 ng	Mortality of larvae were higher after cold stress

**TABLE 2:** Challenges and putative mitigation strategies for the deployment of dsRNA-based forest protection products (FPPs).

Challenges	Putative mitigation
Lack of thorough knowledge of the mechanism of action.	It is essential to know the mechanism of action of dsRNA in target pests to measure the sustainability of the putative control measure. Therefore, dedicated studies on the mechanism of RNAi in forest pests are required. The recent study by Yoon et al. (2018) shows the way forward.
Limited number of forest pest genomes	Sequencing of more forest pest genomes will pave the way for highly efficient and species-specific FPPs.
Low efficiency in key pest species	Appropriate target genes need to be identified for control. Therefore, more studies are required to evaluate potential target genes in forest pest species, and their geographic variability in expression needs to be assessed.
Lack of much information on off-target, non-target effects of dsRNA	The unintended effects caused by dsRNA include silencing of target gene homologs in non-target organisms, off-target silencing of the gene in the target, non-target insects, a saturation of RNAi machinery, and stimulation of immune response. All these effects could influence the performance of natural control agents such as predators, parasites, etc. Hence, the persistence of dsRNA in the forest and the effect of dsRNAs on non-target organisms (NTOs) in the forest ecosystem need to be investigated thoroughly before applying dsRNA inside the forest for pest management.
dsRNA stability	Often low dsRNA stability becomes the major issue. Nanoparticle-based delivery methods can be tested for increasing the efficacy of the dsRNA.
Method of delivery	Considering the vast target area (forest) for application, it is crucial to formulate a cost-effective way to deliver the target dsRNA. Using symbiotic microorganisms such as bacteria or fungi as a potential carrier for dsRNA can be a promising approach.
Higher production and formulation cost	Methods for economical production and formulation of dsRNA need to be developed to compete well with the other commercial insecticides. Chemical synthesis and production of dsRNA in bacteria can pave the way for low-cost production.
The unknown potential for resistance development	Dedicated studies are required to assess the potential for resistance development in target insects as they can achieve resistance to dsRNA by a single mutation, for example, a mutation in the gene coding for proteins involved in dsRNA transport.
Public awareness	The public, forest authorities, and other individual forest owners need to be educated about RNAi technology by organizing public outreach events.

## RNAi Mechanism in Insect Pests: An Overview

RNAi refers to a post-transcriptional gene silencing mechanism prohibiting protein formation by introducing environmental RNA (Fire et al., 1998). Three RNAi pathways have been characterized so far. These are common in insects but not in plants or other animals and include the small interfering RNA (siRNA) pathways, microRNA (miRNA) pathway, and piwiRNA (piRNA) pathway. However, siRNAs are highly sequence-specific to target transcripts, and miRNAs are partial complementarity to target transcripts (Lam et al., 2015; Zhu and Palli, 2020). In contrast to siRNAs and miRNAs, the piRNAs pathway is likely less understood (Farazi et al., 2008).

The RNAi technology application depends on introducing dsRNA into the insect pest body to silence a target gene, subsequently activating the siRNA pathway. Briefly, upon entry of exogenous dsRNA into the cell, the dsRNA is processed into siRNAs by an enzyme ribonuclease III, called Dicer-2. These siRNAs (21–24 nucleotide duplexes) are incorporated in the silencing complex, called the RNA-induced silencing complex (RISC), where the siRNA duplex is unwound. Subsequently, a protein called Argonaute2 (AGO2) cleaves the sense (passenger) strand, and the antisense (guide) strand remains connected with the RISC. Afterward, the antisense strand of the siRNA guides the RISC and allows base pairing to the complementary target mRNA. Subsequently, AGO2 protein degrades or cleaves the target mRNA, and specific post-transcriptional gene silencing occurs (Agrawal et al., 2003; Pecot et al., 2011).

### Variable RNAi Efficiency: What Matters?

A significant degree of variability in RNAi efficiency has been observed between insects and between different orders and between members of the same insect order (Singh et al., 2017; Cooper et al., 2019). In addition, RNAi efficiency can vary among the same transcripts and different areas of the same transcripts, among different transcripts, genotypes, and tissues of the same transcripts (Baum et al., 2007; Zhu et al., 2011; Luo et al., 2013; Zhang et al., 2013; Camargo et al., 2015; Ulrich et al., 2015). For example, coleopterans are more susceptible to RNAi than other insect orders (Terenius et al., 2011; Singh et al., 2017; Cooper et al., 2019; Santos et al., 2021). In Coleoptera, several insects, including the Colorado potato beetle [*Leptinotarsa decemlineata*, CPB] (Zhu et al., 2011; Palli, 2014; Mehlhorn et al., 2021), Western corn rootworm [*Diabrotica virgifera virgifera*, WCR] (Baum et al., 2007; Li et al., 2015b; Fishilevich et al., 2016; Li et al., 2018a), EAB (Rodrigues et al., 2018; Leelesh and Rieske, 2020; Pampolini and Rieske, 2020), and ALB (Rodrigues et al., 2017b; Dhandapani et al., 2020a) have shown a remarkably high sensitivity toward RNAi. In contrast, some other Coleopterans, such as the model insect *Tribolium castaneum* and the African sweet potato weevil [*Cylas puncticollis*], seem less sensitive to RNAi when the dsRNA is administered orally (Prentice et al., 2017). A review by Joga et al. (2016) has discussed several factors contributing to this variability in RNAi sensitivity. These include cellular uptake from the gut environment and dsRNA stability in the digestive system due to enzymatic degradation and a high pH. These factors need to be considered for each target forest pests to ensure the higher efficacy of RNAi.

#### Systemic Properties and dsRNA Uptake

Two types of RNAi are categorized: cell-autonomous RNAi (within a cell) and non-cell-autonomous RNAi (one cell to another and one tissue to another). Environmental RNAi and systemic RNAi together are named non-cell-autonomous RNAi. Environmental RNAi refers to the uptake of exogenous dsRNA by cells in which gene silencing will take place. Whereas systemic RNAi refers to the spread of RNAi signal from one cell to another cell or tissues in the body of an organism (Jose and Hunter, 2007; Whangbo and Hunter, 2008; Huvenne and Smagghe, 2010). For the success of RNAi in any organism, including wood-boring coleopteran forest pests, both environmental RNAi and systemic RNAi should be present and robust.

Two different dsRNA uptake pathways have been illustrated in insects so far. They are the scavenger receptor-mediated endocytosis pathway and the transmembrane Sid-1 channel protein-mediated pathway (Winston et al., 2002; Ulvila et al., 2006; Shih and Hunter, 2011; Wynant et al., 2014a; Cappelle et al., 2016). Several studies have shown that Sid-1 like channel proteins are involved in dsRNA uptake in most insect species, such as the red flour beetle *Tribolium castaneum* (Tomoyasu et al., 2008), the brown planthopper, *Nilaparvata lugens* (Xu et al., 2013), and CPB (Cappelle et al., 2016). In contrast, in dipteran such as the common fruit fly *Drosophila melanogaster*, the dsRNA uptake relies on receptor-mediated endocytosis as this insect lacks *Sid-1* like genes in its genome (Ulvila et al., 2006). Moreover, the number of *Sid-1*-like genes found in the genome of insects varies between insects belonging to different species and orders (Joga et al., 2016). Insects that possess both the transmembrane Sid-1 channel protein-mediated pathway have shown robust environmental RNAi and systemic RNA, for example, *L. decemlineata* (Cappelle et al., 2016). For that reason, CPB is very sensitive to RNAi. However, it was reported that a dsRNA binding protein called Staufen C plays a crucial role in processing the silencing signal and RNAi initiation (Yoon et al., 2018). The lack of Staufen C protein makes lepidopterans less efficient to RNAi.

An enzyme called RNA-dependent RNA polymerase (RdRP) produces the secondary siRNAs by a primer-independent mechanism, amplifying and extending the silencing effect (Schiebel et al., 1993). RdRPs are present in plants and certain eukaryotes, for example, nematodes (Cogoni and Macino, 1999; Mourrain et al., 2000; Smardon et al., 2000; Vaistij et al., 2002). In contrast, no clear RdRP homologs have been found in any insect pest genome so far. It does not mean that insects do not possess a silencing amplification system. RNAi sensitive insects, for instance, coleopterans, have a strong RNAi effect, and the silencing signal lasts for a more extended period. On the other hand, RNAi recalcitrant insects, such as lepidopterans, have moderate or minimal RNAi effect, and the silencing signal is often concise. It indicates that insects have a silencing amplification system, but its mechanism depends on another enzyme with a much similar mechanism as RdRP or depending on another mechanism that has to be discovered (Joga et al., 2016).

The dsRNA uptake by the epithelial cells from the insect gut of the perimicrovillar membrane (PM) is crucial for the success of RNAi as the PM allows the uptake of vitamins, minerals, and insecticidal molecules. However, it is not clear to what extent the PM in the midgut of insect pest functions as a physical barrier to the delivery of dsRNA (Lehane, 1997; Silva et al., 2004; Hegedus et al., 2009; Walski et al., 2014; Liu et al., 2019).

#### Nucleases, Gut pH, and Viruses

Generally, the dsRNA is more stable than the single-stranded RNA, and it should be uptaken by the midgut epithelial cells where the RNAi mechanism will be activated (Katoch and Thakur, 2012). However, salivary nucleases and gut nucleases degrade the dsRNA, limiting the RNAi efficiency (Thompson et al., 2012; Christensen et al., 2013). Christiaens et al. (2014) reported that the ingested dsRNA is degraded rapidly by dsRNases in the salivary secretions and hemolymph of the pea aphid, *Acyrthosiphon pisum*. In addition, Wynant et al. (2014b) reported that the dsRNA degraded in the midgut juices of the pest desert locust, *Schistocerca gregaria*. Similar observations were done by Taning et al. (2016) with the Asian fruitfly *Drosophila suzukii* and southern green stinkbug *Nezara viridula* by Sharma et al. (2021). Fortunately, RNAi susceptible insects often showed less degradation of dsRNA by dsRNAses.

The pH in the gut of insects has been found to vary between insects belonging to different orders, for example, high acidic in Coleopterans and high alkaline in few Lepidopterans. The alkaline nature of the midgut plays an important barrier for the delivery of dsRNA in Lepidopteran species and provides an unfavorable environment for ingested dsRNA (Dow, 1992; Kolliopoulou et al., 2017). Furthermore, the existence of viruses may also play a critical obstacle for RNAi efficiency as the viruses can saturate RNAi core machinery (Kanasty et al., 2012) and development of RNAi-blocking proteins called viral suppressors of RNA silencing (VSRs) (Haasnoot et al., 2007). Hence, all such possibilities need to be evaluated in the target forest pests.

#### Length and Concentration of dsRNA

The length and the concentration of exogenous dsRNAs are significant for the success of RNAi. However, the requisite length of dsRNA varies among insect pests (Bolognesi et al., 2012). For example, Miller et al. (2012) reported that 60 base pairs length of dsRNA induced 70% gene knockdown and 30 base pairs length of dsRNA induced 30% gene knockdown in *T. castaneum*. This study clearly explains that long dsRNAs are required for effective RNAi in insects. However, many studies reported that effective RNAi was seen when 140 to 500 -bp dsRNAs were used (Huvenne and Smagghe, 2010). Whereas the short length dsRNAs are preferred to induce the target-specific gene silencing to minimize the off-target and non-target effects.

For effective RNAi, optimal concentration has to be determined for every target gene in the forest pests. For example, Baum et al. (2007) demonstrated that dsRNA targeting the V-ATPase gene caused silencing in the WCR in a concentration-dependent manner. However, achieving higher silencing by exceeding optimal concentration is not true (Meyering-Vos and Müller, 2007; Shakesby et al., 2009). Moreover, introducing multiple dsRNAs of different target genes into the insect body may lead to poor RNAi efficiency due to competition among introduced dsRNAs during cellular uptake (Parrish et al., 2000; Barik, 2006; Miller et al., 2012).

## Prospects and Challenges for RNAi Against Forest Pests

**FIGURE 2:**
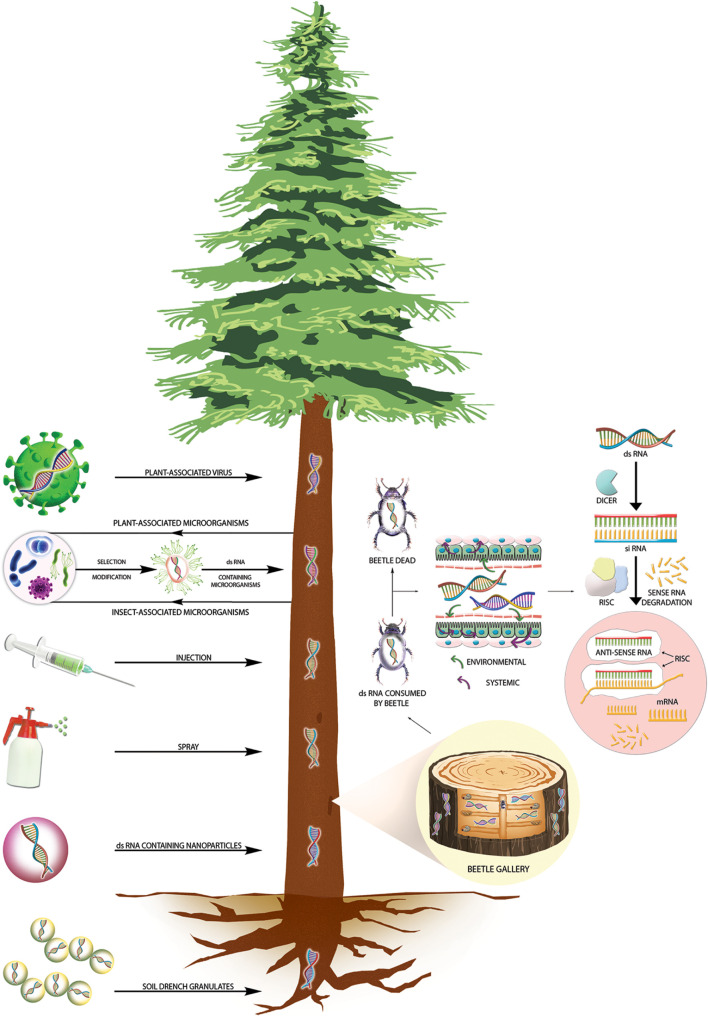
RNAi-mediated plant protection from insect pests at the tree level. The left panel shows different transformative and non-transformative dsRNA delivery strategies to control wood-boring insect pests at the tree level. A section magnified in the right panel shows dsRNA in both vessel systems (xylem and phloem), allowing dsRNA to move up and down and reach the insect galleries. Upon ingestion of dsRNA by wood-boring coleopterans, the dsRNA will reach the gut epithelial cells, where the cellular siRNA mechanism of gene silencing will be initiated, which will lead to target insect death.

Wood-boring insects thrive inside the bark, trunk on a nutritionally limiting diet. They tunnel in the inner layer where water and nutrients are available. They attack either healthy or weakened and dead trees based on their statuses like primary invader or secondary invader. It is worth mentioning that primary invaders (i.e., *Ips typographus*, Eurasian spruce bark beetle) mostly kill the infested tree. However, most often, the damage caused by an infestation of wood borers remains unnoticed until the tree showed visible symptoms or external signs of damage, such as the entry hole of a wood borer or sawdust (Hlásny et al., 2019). This hidden lifestyle of wood borers, contrary to most other agricultural pests, causes considerable impediments for control measures, even for RNAi-based FPPs. Perhaps choosing a suitable strategy to deliver dsRNA is a big challenge in RNAi-based forest protection methods (Table 2). Several possible dsRNA application strategies are available that can be deployed against forest pests. They are transiently transformative (recombinant symbiont or virus) and non-transformative (nanoparticles, trunk injections and spraying, root soaking, and soil drench) methods (Figure 2). Due to the transient feature of the molecules used in non-transformative delivery methods, target pests have limited exposure to the dsRNA molecules, delaying the resistance development. Deploying the RNAi based Plant-incorporated protectants (RNAi-PIPs) via transgenic trees (transformative approach) seems to be a less viable solution against wood-boring forest pests due to public and scientific concern (i.e., gene flow), lack of suitable tree transformation protocols, high development time and cost, and extensive regulatory processes (Cagliari et al., 2019). Hence, in the present review, we omitted the discussion on the transformative approaches. However, it is worth mentioning here that researchers already developed efficient and stable plastid transformation protocols for poplar, which can be considered for developing RNAi-PIPs against pests infesting the green tissues of poplar (Wu et al., 2019).

### Selection of Target Gene

The success or failure of RNAi technology experiments mainly depends on the selection of the target gene. For successful gene silencing, selection of target genes and target regions within genes, its expression pattern (developmental and tissue-specific), the insect species and/or population, and the structure and sequence of the dsRNA are crucial. The second-generation sequencing, genome-wide screens, existing data from closely related species, tissue, and developmental stage-specific expression profiles, and gene ontology are valuable tools that can provide information on target gene selection and screening but require more extensive resources to perform (Wang et al., 2011; Knorr et al., 2018). The ideal target gene should have a high transcription rate and produce a protein with a low half-life, and transcription reduction of the intended target gene must cause mortality in the pest insect (Scott et al., 2013; Cooper et al., 2020). After candidate gene selection, dsRNA-induced mortality screening in multiple life stages is required to assess the desired phenotype induced by specific dsRNA. Often it is worth starting screening multiple genes for putative candidate selection for RNAi application. Moreover, the stage and tissue-specific expression levels of core genes in the RNAi machinery must be evaluated to achieve the optimum window for RNAi application against target forest pests (You et al., 2020). It will be optimal to follow up the RNAi studies via RNAiSeq studies to evaluate the changes in the expression of other genes impacted by the RNAi treatment (Oppert and Perkin, 2019; Xu et al., 2021). Furthermore, based on the recent report, it will be optimal to evaluate any synergistic engagement of the resident gut microbiome in the dsRNA-induced mortality of target insects (Xu et al., 2021). Such approaches will lead to a better understanding of the mechanism of action of RNAi-based FPPs and facilitate the enhancement of their sustainability.

#### RNAi for Disrupting Pest Communication

The main objective of RNAi-based FPPs is to reduce the forest pest population level below the epidemic level. Pheromone is a species-specific chemical substance for insect communication. The RNAi can be deployed to disturb the pest’s reproductive behavior by silencing genes involved in producing sex pheromones. For instance, *Helicoverpa armigera* could not find the female moths when two pheromone-binding proteins were silenced by RNAi, which decreased mating behavior (Dong et al., 2017). Similarly, genes involved in pheromone production in bark beetles can be targeted via RNAi to disrupt communication, such as aggregation pheromone signal for a mass attack in *Ips typographus*. The delivery of dsRNA through trunk injection and/or soil drench molecules may move through the phloem, and the pheromone-binding proteins will be silenced in the beetle upon phloem-feeding (Figure 1).

### Design of Species-Specific dsRNA

**FIGURE 3:**
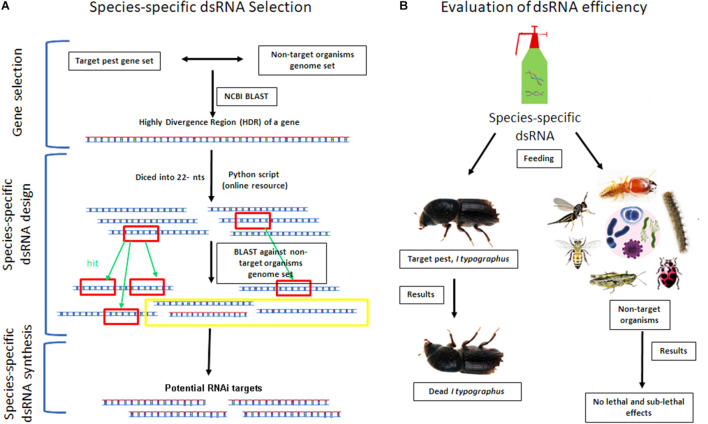
Mitigation strategies of RNAi-based pesticides to overcome non-target effects. Schematic illustration of *in silico* analysis of species-specific dsRNA selection **(A)** and evaluation of dsRNA efficiency against the target pest and non-target organisms **(B)**. The selection of a target gene is a crucial step for the success of RNAi. For that, the Highly Divergence Region (HDR) of a target gene will be identified by doing NCBI BLAST of target pest gene set against the closely related non-target organism’s genome set. Subsequently, the HDR of a gene will be diced into all possible 22-nt stretches by using a python script (Online resource) (Taning et al., 2021). Afterwards, these generated 22-nt sequence stretches will be used as a query in a BLAST search against the non-target organism’s genome set for complementary sequences (hits). Finally, the sequence-specific dsRNA synthesis will be done for the identified distinct sequence region from the target gene **(A)**. Then, the sequence-specific dsRNA will be exposed to the target forest pest (i.e., *Ips typographus*) and other non-target organisms (i.e., honeybees, wasps, butterflies, earthworms; termites, and plant-growth-promoting and soil organisms). We hypothesize that only the target pest will be affected upon feeding with no lethal and sub-lethal effects on non-target organisms **(B)**. The species-specific dsRNA product can be commercially available for forest application after evaluation of all biosafety measures.

The optimal length of dsRNA uptake varies from insect to insect, and previous reports showed that this optimum lies between 200 and 520 bp (Huvenne and Smagghe, 2010; Bolognesi et al., 2012). Recent mutagenesis analysis revealed dsRNA with more than 80% sequence complementarity with the target gene substantially triggers the RNAi effect (Chen et al., 2021). A dsRNA containing ≥ 16 bp regions of perfect complementarity or >26 bp regions of nearly matched sequence with one or two mismatches barely distributed (i.e., single discrepancy placed between ≥ 5 bp complementary region or incompatible couplets inserted between ≥ 8 bp complementary regions) also trigger RNAi mediated gene silencing (Chen et al., 2021). A similar finding was also documented about RNAi against plant sap-feeding hemipteran pests indicating maximum dsRNA sequence complementarity was crucial for RNAi-based gene silencing in related hemipteran species (Arora et al., 2021). Additionally, most of the genes are not stably expressed during the life cycle of the insect, and the dosage of dsRNA concentration should be adjusted according to the abundance of target mRNA. The concentration should be species-specific as the RNAi efficiency is less where dsRNAses are more (i.e., insect gut), and in such cases, an overdose of dsRNA may be required to induce the desired knockdown of target genes (Scott et al., 2013). Considering such parameters to estimate off-target effect in the available target and non-target organism genome using bioinformatics tools (i.e., genome-wide blast analysis), dsRNAs can be designed with higher efficiency and species specificity. For instance, targeting segments (i.e., >100 bp with no contiguous stretches of sequence identity more than 20 bp) from orthologous genes with high divergence regions (HDRs) or homologous genes with HDRs or genes that are lost in all closely related species can be a good starting point for securing species-specific targets for RNAi (Figure 3A). Advanced bioinformatics pipelines can be prepared to find such targets in the forest pest genomes for screening. Hence, having high-quality forest pest genomes and transcriptomes (Table 3) is also critical in designing species-specific dsRNA (Table 1).

**TABLE 3:** List of genome and transcriptomics studies done with wood-boring coleopterans.

S.No.	Species	Common name	Genome		Transcriptome		References/source
			Available	Accession No.	Available	Accession No.	
1	*Ips typographus*	European spruce bark beetle	**✓**	PRJNA671615	**✓**	PRJNA702426; PRJNA679450; and PRJNA178930	Andersson et al., 2013; Powell et al., 2020
2	*Ips pini*	Pine engraver beetle	**✕**		**✓**	PRJNA90755; PRJNA87977; and CB407466–CB409136	Eigenheer et al., 2003; Keeling et al., 2006
3	*Dendroctonus ponderosae*	Mountain pine beetle	**✓**	PRJNA360270; PRJNA162621 and PRJNA179493	**✓**	PRJNA37293; PRJNA189792; PRJNA189795; PRJNA269763; PRJNA203305; and PRJNA317010	Aw et al., 2010; Keeling et al., 2012, 2013, 2016; Robert et al., 2013; Nadeau et al., 2017
4	*Dendroctonus frontalis*	Southern pine beetle	**✕**		**✓**	PRJNA79903	DOE Joint Genome Institute Dendroctonus frontalis EST project
5	*Dendroctonus armandi*	Chinese white pine beetle	**✓**	PRJNA530572	**✕**		Godefroid et al., 2019
6	*Dendroctonus valens*	Red turpentine beetle	**✕**		**✓**	PRJNA656966	Zhao et al., 2021
7	*Trypodendron signatum*	Ambrosia beetle	**✓**	PRJNA418542	**✕**		Trypodendron signatum RefSeq Genome
8	*Tomicus yunnanensis*	Yunnan pine shoot beetle	**✕**		**✓**	PRJNA362869; PRJDB2098; PRJDB746; PRJNA396694; and PRJNA175397	Zhu et al., 2012a, b
9	*Agrilus planipennis*	Emerald ash borer	**✓**	PRJNA230921; and PRJNA343475	**✓**	PRJNA271706; PRJNA263193; PRJNA222581; PRJNA79619; PRJNA173782; and PRJNA508756	Lowe and Eddy, 1997; Lord et al., 2016
10	*Anoplophora glabripennis*	Asian longhorned beetle	**✓**	PRJNA348318; PRJNA167479; PRJEB3278; and PRJNA167479	**✓**	PRJNA196436; PRJNA274806; PRJNA299040; PRJNA395783; PRJNA613658; and PRJNA691113	Lowe and Eddy, 1997
11	*Euwallacea fornicatus*	Polyphagous shot-hole borer	**✓**	MT897842	**✓**	PRJNA260703	Wang et al., 2020a

### Evaluation of Off-Target and Non-target Effects

To minimize the off-target effects and avoid cross silencing, the dsRNA constructs should be chosen in non-conserved regions of other species or isoforms of the target gene (Figure 3B). The previous studies have shown that the well-designed dsRNA construct can be highly species-specific. For instance, the 3′-UTR region targeted γ-Tubulin transcript showed species-specific knockdown in four closely related *Drosophila* species (Whyard et al., 2009). Similarly, Kumar et al. (2012) used three highly similar CYP genes and showed particular transcript reductions in the Tobacco hornworm, *Manduca sexta*. Very recently, Pampolini and Rieske (2020) had evaluated the effect of EAB-dsRNA targeting heat shock 70-kDa protein (*hsp)*, shibire *(shi), and* U1 small nuclear ribonucleoprotein *(sn-rnp)* against non-target organisms (NTOs) such as CPB (Coleoptera: Chrysomelidae); the spotted lady beetle *Coleomegilla maculata* (Coleoptera: Coccinellidae); the eastern subterranean termite, *Reticulitermes flavipes* (Blattodea: Rhinotermitidae); honeybee, *Apis mellifera* (Hymenoptera: Apidae); *Tetrastichus planipennisi* adult (Hymenoptera: Eulophidae), and *Spathius galinae* (Hymenoptera: Braconidae). Results did not suggest any adverse effect on the NTOs challenged against EAB-dsRNAs. However, such studies evaluating the RNAi against NTOs are scarce in the field of forestry and need to be incorporated regularly in the experimental plan for developing RNAi-based forest protection products (FPPs).

### Enhancing the Stability of dsRNA for Environmental Application

Before RNAi-based non-transformative products are applied against forest pests, the obstacles of insufficient RNAi sensitivity (if present) or quick environmental degradation possibility (i.e., after tropical application, trunk injection, and root absorption) must be resolved. An elegant solution could be dsRNA packaging that protects dsRNA from degradation and facilitates selective uptake in the target tissue. It may be achieved via developing novel delivery systems. However, the specificity of these systems has to be prudently evaluated. Therefore, the effect of all proposed delivery strategies on NTOs needs to be thoroughly evaluated before applying FPPs.

## Novel Delivery Methods Relevant to Forest Pest Management

### Nanoparticles as Protectors of dsRNA

The nanoparticles, typically one to a few hundred nanometers in size, are formed by encapsulating dsRNA with organic and inorganic materials. These nano-complexes have shown significant promise to overcome the obstacles for enhancing RNAi efficiency in many insect species by improving dsRNA absorbance, stability, protecting dsRNA from salivary and gut nucleases, and improving cellular uptake efficiency (Christiaens et al., 2020b; Yan et al., 2020b, 2021; Zhu and Palli, 2020). The chitosan-derived nanoparticles are widely used, biodegradable and non-toxic (Dass and Choong, 2008; Gurusamy et al., 2020a; Lichtenberg et al., 2020). Chitosan nanoparticle binds the silencing signal dsRNA/siRNA through electrostatic interaction (Zhang et al., 2010). Christiaens et al. (2018) reported that *chitin synthase B* targeting dsRNA delivered through guanylate polymers given protection to silencing signals from the gut nucleases, which led to increased mortality in the RNAi recalcitrant pest such as beet armyworm (*Spodoptera exigua)*. However, nanoparticles with higher molecular weight may give better binding efficiency to dsRNAs/siRNAs, but their solubility may decrease, giving poor cellular uptake (Baigude and Rana, 2009). Nanoparticle encapsulation is proven highly effective in controlling many insects and may be used against forest pests via GMO-free approaches such as trunk injection or root absorption methods (Figure 2). Recent discoveries also indicated possibilities for a nanocarrier-mediated transdermal dsRNA delivery system to enhance RNAi efficiency after spraying (Zheng et al., 2019; Yan et al., 2020a).

### Liposomes and Lipid-Based Transfection Reagents Enhancing Cellular Uptake

The delivery of dsRNA through liposomes would be a promising strategy as liposomes are made up of natural lipids, non-toxic and biodegradable (Van Rooijen and van Nieuwmegen, 1980). The dsRNA-encapsulated liposomes get into the cell’s cytoplasm both by endocytosis and fusion with the plasma membrane. As already discussed in the above text that some insects possess dsRNA uptake through receptor-mediated endocytosis only, and it is a prolonged process, and transfection reagent is required for the efficient cellular uptake of dsRNA (Saleh et al., 2006; Ulvila et al., 2006; Whyard et al., 2009). Taning et al. (2016) reported that effective RNAi was seen in the spotted-wing *Drosophila* (*Drosophila suzukii*) when the specific gene targeting dsRNA is delivered through liposomes. The transfection reagents are lipid-based products, and the dsRNA-transfection reagent complexes enhance the RNAi efficiency in insects by protecting the dsRNAs from endonucleases, aiding the dsRNA uptake into the insect cells, and escape of dsRNAs from endosomal compartments within the cells (Cooper et al., 2019; Christiaens et al., 2020b; Gurusamy et al., 2020b). The previous studies used different transfection reagents on insect species from different orders, including Diptera (*Drosophila* spp., *Ae. aegypti*), Hemiptera (Neotropical brown stinkbug, *Euschistus heros*), Blattodea (German cockroach, *Blattella germanica*), and Lepidoptera (fall armyworm, *Spodoptera frugiperda*) (Christiaens et al., 2020b). Karny et al. (2018) observed the translocation of liposome nanoparticles from leaves to roots. They used liposomes containing a fluorescent tracer (fluorescein, green) and applied foliar on cherry tomato plants. After 72 and 96 h post application, secondary and tertiary roots were collected and imaged using confocal microscopy. For 72 h, the particles gradually accumulated in individual root cells and after 96 h, the liposomes disintegrate and release their cargo into the cytoplasm. The light, pH, temperature, enzymatic condition, and oxygen mainly affect the stability of liposomes (Wang et al., 2020b). In our lab, for *in vitro* studies, we used liposomes or proteinaceous carriers for protecting the dsRNA from the enzymatic degradation and enhances the dsRNA uptake by cells and these results seem very promising. Although there are no reports on the delivery of dsRNA through liposomes and transfection reagents on the crop to suppress insect pests so far, they can be considered carriers of RNAi against forest pests.

### Proteinaceous dsRNA Carriers Facilitating the Uptake

The negatively charged plasma membrane acts as the main barrier to the uptake of negatively charged dsRNAs. Cell-penetrating peptides (CPPs) transports the silencing signal dsRNAs/siRNAs and facilitates the uptake of the silencing signal into the gut epithelial cells (Milletti, 2012). Chen et al. (2012) and Zhou et al. (2015) reported that CPPs were successfully internalized hormones and plasmid DNA in insect cells. Gillet et al. (2017) reported that the gene *chitin synthase II* was successfully knockdown in the cotton boll weevil (*Anthonomus grandis*) when this pest fed on dsRNA pairing with the peptide-transduction domain (PTD) and a dsRNA-binding domain (dsRBD). Peptide-dsRNA complexes fed with early instar larvae of red flour beetle, *Tribolium castaneum* showed mortality (Avila et al., 2018). Additionally, feeding fluorescently tagged dsRNA peptide capsules were distributed throughout the red flour beetle, *Tribolium castaneum* tissues but fluorescently tagged dsRNA alone did not show widespread dispersal. In plant cells, delivery of dsRNAs has to face two barriers, i.e., cell wall and cell membrane. Several studies used polymer-based carriers to deliver plasmid DNA and proteins into intact plant cells and suggested that these systems deliver interfering RNAs (Martin-Ortigosa et al., 2012; Chang et al., 2013; Hussain et al., 2013; Demirer et al., 2019). However, few studies reported that nanoparticles were used for delivering RNAi molecules into the plant cells (Demirer and Landry, 2017; Mitter et al., 2017). The dsRNA conjugated with layered double hydroxide clay nanosheets (BioClay) was sprayed on *Nicotiana tabacum* and detected the dsRNA up to 30 days (Mitter et al., 2017). Demirer et al. (2019) used single-walled carbon nanotubes to improve the cellular delivery of siRNAs into *Nicotiana benthamiana* plants and observed that the polymeric carrier protects the siRNA against degradation RNaseA. The peptide-based carrier systems were used for rapid and efficient RNAi-mediated gene silencing in diverse plant species (*Arabidopsis thaliana*, *Nicotiana benthamiana*, *Solanum lycopersicum*, and poplar), *N. tabacum* suspension-cell cultures, and rice callus tissue (Unnamalai et al., 2004; Numata et al., 2014, 2018). More recently, Martinez et al. (2021) reported designing a lectin-based dsRNA delivery system by fusing a dsRNA-binding domain (dsRBD) to the GNA lectin domain. This GNA lectin-dsRBD fusion protein improved the cellular uptake of dsRNA in a midgut cells line and increased insect mortality for dsRNA-v-ATPase-A. Hence, De Schutter et al. (2021) reviewed the boosting of dsRNA delivery in plant cells with peptide and polymer-based carriers for an increased crossing of the plant cell wall, allowing efficient environmental RNAi in plants and improving the RNAi response in pest control. Such observations proved that CPPs or aliphatic peptide capsules could enhance RNAi efficiency and be considered against forest pests showing less sensitivity to RNAi treatments.

### Root Absorption and Trunk Injection for Big Trees

It is known that phloem transports food, organic material, and the silencing signal, whereas the xylem is considered the channel for minerals and water movement (Buhtz et al., 2008). The dsRNAs or siRNAs are stable in the phloem where the environment is RNase-free (Doering-Saad et al., 2002). Once the dsRNA reaches the tissue, the silencing signal can spread to the adjacent cells (Melnyk et al., 2011). However, persistency and continuous supply of dsRNA are needed for the success of RNAi. Therefore, the dsRNA delivery through irrigation and trunk injection could be a more promising strategy than the foliar spray to control wood-boring insect pests (Wise et al., 2014; Ghosh et al., 2018; Berger and Laurent, 2019). With these strategies, suppressing all kinds of insects, like, chewing, piercing-sucking, root grubs, and wood-boring forest pests, would be possible as the exogenous dsRNA will reach every part of the plant (Andrade and Hunter, 2016). For example, Hunter et al. (2012) reported that the dsRNA is stable in the plants for 57 days, and siRNAs are detected in plants for almost 4 months when the citrus plants are drenched in dsRNA solution.

Moreover, the dsRNA is stable only for 5–8 days in leafhoppers and psyllids when fed on dsRNA-treated citrus plants. Additionally, Li et al. (2015a) also reported high mortality of the Asian corn borer, *Ostrinia furnacalis*, when dsRNA targeting Kunitz-type trypsin inhibitors (dsKTI) irrigated to maize seedlings. Finally, a recent confocal microscopy study provided evidence of EAB-dsRNA absorption via plant tissue, indicating the feasibility of dsRNA delivery against forest pests such as EAB (Pampolini et al., 2020).

Alternatively, by trunk injections, dsRNAs can deliver directly into a tree’s phloem where the companion cells do not have a nucleus, and the silencing signal rapidly spreads toward the shoots and roots; and the silencing signal will last for a more extended period. Already several companies manufactured injectors such as Arborjet^®^ (Joga et al., 2016) to deliver dsRNAs through trunk injections into trees. Dalakouras et al. (2018) showed that hairpin RNAs (hpRNAs) against *M. domestica* injected in *V. vinifera* plants via trunk injection was efficiently translocated and restricted to the xylem vessels and apoplasts, so the plant dicer-like (DCL) endonucleases were unable to process the hpRNAs and injected RNA molecules were stable for at least 10 days after post-application. These innovative methods may have a significant impact on RNAi-mediated forest protection. However, injecting each tree in the forest is not a sustainable solution.

### Microorganisms as Carriers: Potential for Forestry Application

#### Bacteria for Minimizing Production Cost and Delivering dsRNA

A persistent and large amount of dsRNAs is required for effective RNAi in forestry applications. The commonly used and recently identified *Escherichia coli* strains, i.e., *HT115*(DE3) and pET28-*BL21*(DE3), contains the deletion of the RNase III gene (*rnc*) with a T7 expression vector that can be used to produce higher concentrations of dsRNA with less cost (Timmons et al., 2001; Ma et al., 2020). Apse RNA Containers^TM^ (ARCs) technology developed by a biotechnology company allows the production of dsRNAs in large volumes using bacteria. Plasmids coding for proteins such as capsids is co-transformed with another plasmid coding for dsRNA sequences plus a “packing site.” While bacteria grow in culture, they make protein subunits self-assembled around RNA encompassing the packing site sequences. After purification of the engineered bacteria, the resulting RNA is environmentally stable and a ready-to-spray product (Joga et al., 2016). The production of bacterial-expressed dsRNA is inexpensive compared with *in vitro* production (Huvenne and Smagghe, 2010).

The delivery of dsRNA through bacteria is more beneficial than dsRNA delivery through spray and trunk injections. Dhandapani et al. (2020b) reported that when specific gene targeting dsRNA delivered through bacteria to the Asian long-horned beetle (*A. glabripennis*) has seen high efficiency of RNAi. The dsIAP and dsActin were combinedly expressed in the *HT115*(DE3) strain, and the heat-killed bacteria were sprayed on potato plants, which protected the plants from CPB damage (Máximo et al., 2020). The *HT115*(DE3) was used to express dsRNAs targeting the arginine kinase gene in South American tomato pinworm, *Tuta absoluta*, and the dsRNA expression expressed that heat-killed bacteria were spread on artificial media caused 70% larval mortality (Bento et al., 2020). The dsRNAs are expressed in heat-killed bacteria specific to the target genes SRP54 and actin fed to *Plagiodera versicolora* (Coleoptera: Chrysomelidae) caused significant mortality (Zhang et al., 2019). The dsHvSnf7 was expressed in heat-killed bacteria and sprayed on detached leaves, and plants showed significant mortality in *Henosepilachna vigintioctopunctata* (Coleoptera: Coccinellidae) larvae (Lü et al., 2020). More recently, researchers used endogenous symbionts like *Rhodococcus rhodnii* for expressing the dsRNAs, specifically targeting many different genes and observed phenotypes in insect species (Whitten et al., 2016). Researchers identified symbionts from both kissing bug (*Rhodnius prolixus*) and western flower thrips (*Frankliniella occidentalis*) that can be engineered to deliver dsRNA. Expressed dsRNAs symbionts administered orally, resulting in the suppression of target genes in insect species. The above research suggests that the symbiont mediated dsRNA delivery method may be viable in specific cases where symbionts can be transferred between individuals, making it less costly and efficient for forest pest management. Furthermore, symbiont mediated RNAi (SMR) gives two levels of specificity through carrying species-specific dsRNA by the species-specific symbionts.

#### Viruses Inducing and Delivering dsRNA

Plant infecting viruses move through the phloem systematically. Therefore, controlling insect pests through recombinant viruses would be a promising strategy. Virus-induced gene silencing (VIGS) is a method for enhancing RNAi efficiency in insects in two ways, i.e., either adding or replacing a gene in the virus and the target dsRNA is enclosed in viral capsid proteins called virus-like particles so that the recombinant virus will produce the desired dsRNA during replication specific to the target pests (Kolliopoulou et al., 2017, 2020). Insects viruses are species-specific, for example, baculoviruses, and these viruses are could be engineered to express specific target gene dsRNAs, subsequently delivered to the field to control insect pests (Swevers et al., 2013). *Nicotiana benthamiana* plants infected with recombinant tobacco mosaic virus expressed dsRNAs specific to the chitinase 1 or 2 genes of oriental armyworm, *M. separata*, and the larvae showed suppression in chitinase gene expression within gut tissues and reduced body weight (Bao et al., 2016). Wuriyanghan and Falk (2013) reported that the potato psyllid, *Bactericera cockerelli* successfully controlled by delivering dsRNA against *actin* and *V-ATPase* through the recombinant *Tobacco mosaic virus* (TMV). For that reason, this technique could be useful to control forest pests, including bark beetles. Perhaps more research is needed for the application of VIGS against coleopteran wood-boring forest pests. The VIGS mediated RNAi technology is still restricted to the lab experiments and no reports so far for forest insect pest management under field conditions. However, in a forest, the infested trees will be identified and subsequently applied VIGS through trunk injection. However, it is very difficult to apply VIGS in the environment due to the strict regulations and environmentalists, and it seems very difficult after the Covid pandemic.

#### Fungi as dsRNA Carrier

Fungal-induced gene silencing (FIGS) technology also we can use to enhance RNAi in forest pest management. Generally, the FIGS system can develop in two main ways, i.e., dsRNA can express in common fungi (*Saccharomyces cerevisiae* or *Pichia pastoris*) and entomopathogenic fungi that will enhance RNAi efficiency in insects (Van Ekert et al., 2014; Chen et al., 2015; Hu and Wu, 2016; Hu and Xia, 2019; Mysore et al., 2019). Yeasts mediated dsRNA delivery caused significant mortality and delayed larval development in dipteran insects like *Ae. aegypti* (Van Ekert et al., 2014; Mysore et al., 2019). Likewise, the spotted-wing drosophila, *Drosophila suzukii* larval survival rate, and the egg-production rate were decreased drastically when dsRNA targeting *y-tubulin 23C* (*yTub23C*) was administered orally through the yeast *Saccharomyces cerevisiae* (Murphy et al., 2016). The dsTLR7 expressed entomopathogenic fungi, *Isaria fumosorosea*; consumption caused mortality up to 40% in second-instar *B. tabaci* nymphs (Chen et al., 2015). Similarly, the entomopathogenic fungi *Metarhizium acridum* were used to express dsRNAs targeting the α and/or β subunit genes of the locust F1F0-ATP synthase caused mortality in *L. migratoria* larvae (Hu and Xia, 2019). Recently, colleagues from the United States have started transforming bark beetle-associated yeast *Ogataea pini* to produce target dsRNA against bark beetles *Ips calligraphus* (source: personal communication). Hence, the FIGS technology is already under consideration for forest protection, but further optimization is required.

#### Microalgae or Lichens

Symbiotic interaction sustains expanded consideration among all parts of science because it helps to build the unifying themes across ecological, evolutionary, developmental, semiochemical, and pest management hypotheses. For example, Klepzig et al. (2009) reviewed the symbiotic relationship of bark beetles with fungus, bacteria, viruses, protozoa, and algae. Lichens are symbiotic organisms made up of a fungus and green algae or cyanobacterium growing jointly on the trees with multiple forms and colors. They provide food, cover, and nesting materials for a variety of insects like bristletails, barklice, katydids, grasshoppers, webspinners, butterflies, moths, moth larvae, lacewing larvae, mites, spiders, and many beetles (Speer and Waggoner, 1997; Edgerly and Rooks, 2004). Although commonly lichens are grouped into fruticose (branched or tubed), foliose (flattened or leafy), and crustose (crusty), most are pale green, brownish-green, orange, and yellow. In 2012, the University of Wisconsin reported that the lichen moth larvae (*Hypoprepia* sp.) eat lichens and blue-green algae that they find growing on tree trunks. In England, psocid species eat *Lecanora conizaeoide* lichens on larch trees randomly or only eat apothecia. For example, the *Campecopea hirsuta* feeds only algal cells on *Lichina pygmaea* (Wieser, 1963). In Australia, the web-spinner insects, i.e., *Notoligotoma hardyi*, preferred lichens as a food (Gressitt et al., 1965). Mites have a wide range of feeding habits, i.e., feed on dead plant materials, algae, and lichens (Walter and Proctor, 1999). A recent review revealed the potential use of lichen solvent extracts and metabolites as an insecticidal agent against various pests causing damage to plants, especially coleopterans (e.g., Sitophilus and Leptinotarsa) and insect vectors transmit dreadful diseases to humans such as Aedes, Anopheles, and Culex (reviewed by Sachin et al., 2018).

The marine algae (*S. wightii* and *P. pavonica*) extracts are used as an eco-friendly nymphicide or biopesticide for controlling the *Dysdercus cingulatus* (Fab.) (Hemiptera: Pyrrhocoridae) in cotton pest management (Asharaja and Sahayaraj, 2013). The mosquito larvae are aquatic and eat microalgae via filter feeding because microalgae grow within the larval habitat, and it is an excellent choice to provide direct exposure to dsRNA by engineered microalgae. Based on this idea, researchers developed engineered microalgae (*Chlamydomonas reinhardtii*) that express dsRNA specific to the enzyme 3-hydroxykynurenine transaminase fed to the *Anopheles stephensi* mosquito larvae showed 53% mortality (Kumar et al., 2013). Therefore, further research is needed for microalgae-mediated RNAi in forest insects, i.e., identifying and selecting suitable symbiont microalgae for forest insects, expressing desired dsRNAs for suitable target genes, and enhancing RNAi efficiency in forest insects.

## Biosafety Considerations for FPPs

RNAi has proved the potential to suppress pests and save beneficial insects from diseases and parasites. For example, a non-target organism screening with the dsRNA-based biocontrol product targeting *L. decemlineata* revealed the selectivity and safety of the dsRNA sequence even for closely related species and beneficial insects (Bramlett et al., 2020). Similarly, a genome-wide off-target screen in important bumblebee pollinators of *Bombus terrestris* with dsRNA targeting pollen beetle αCOP revealed no reduction in the transcript level for all putative off-targets, including an off-target with a 20-continuous-nucleotide match (Taning et al., 2021). Also, for a set of potential targets in the EAB, off-target effects were screened. After confirming the dsRNA’s specificity, they are qualified as potential targets to suppress EAB populations (Rodrigues et al., 2018). However, deploying RNAi-based FPPs presents unique challenges for ecological, environmental, and human risk assessments.

In our opinion, for forestry application, safety assessments should include evaluating environmental safety for the NTOs (Figure 3B). Due to its rapid environmental degradation, the trees treated with the exogenous applications of dsRNAs to control forest pests will not be considered genetically modified organisms (GMOs) (Shew et al., 2017; Arpaia et al., 2021). However, it is significant to follow biosafety assessments for FPPs before deployment. The noticeable effects on non-target organisms exposed to RNAi will provide significant evidence for ensuring safety. Bioinformatics tools perform an essential role in the development of species-specific targets (Figure 3A). However, the availability of a limited number of genome sequences from forest pests limits risk estimates. Furthermore, the effects of dsRNA on soil and other plant-associated beneficial microorganisms need to be evaluated to measure the effect of dsRNA in forest ecosystems accurately (Figure 3B).

The deployment of genetically engineered bacteria, fungi, or viruses capable of delivering the RNAi-based FPPs in the forest needs to be under some regulatory framework as they will also be considered GM products. Unfortunately, there is no risk assessment protocol for genetically engineered microorganisms delivering dsRNA (in short, RNAi-microbes) so far as RNAi-based GM crops (Papadopoulou et al., 2020). One putative reason is that the deployment of RNAi-microbes is an intriguing idea (i.e., SMR) that has just begun to blossom (Zhang et al., 2019). Regulatory agencies worldwide need to devise the environmental risk assessment protocol dedicated to deploying RNAi-microbes in the forest. In our opinion, RNAi-microbes can be evaluated for their mechanism of action, specificity (including the designing the dsRNA), active ingredient, environmental fate, ecotoxicology [impact on humans, other NTOs (including soil organisms), other microbes in the microhabitat, soil and water], Toxicity-Exposure-Ratio (TER), off-site movement, the requirement of the periodic application, immune response and resistance management in target pest while formulating the legal framework relating to the risk of dsRNA-based FPPs. Bioinformatics tools can assist in the selection of surrogate species for tiered toxicology testing. Nevertheless, RNAi pesticides occur naturally inside target organisms and thus a potentially safer alternative to synthetic pesticides.

## Conclusion and Future Perspectives

RNAi is a robust technology that can bring a new paradigm in forest pest management, but some obstacles yet limit its implementation. Researchers are developing a variety of methods for boosting RNAi in wood-boring coleopterans, i.e., selection of appropriate target genes by using sequencing data, genetic modification of microbes and plants, identifying the components of extracellular vesicles, dsRNA complexation/encapsulation with nanomaterials (Sinisterra-Hunter and Hunter, 2018; Cooper et al., 2019; Christiaens et al., 2020a). Furthermore, forest protection with RNAi-based pesticides would be a novel integrated pest management strategy (IPM) due to its high sequence-dependent specificity and better safety than conventional pesticides (Wang et al., 2016; Mitter et al., 2017; Christiaens et al., 2020a; Taning C.N. et al., 2020). Hence, the researchers are focusing on controlling forest pests by using this technology, and its potential to control several coleopteran wood-boring forest pests such as bark beetles, EAB, ALB is already experimentally proven (Rodrigues et al., 2017a, b, 2018; Kyre et al., 2019, 2020; Dhandapani et al., 2020a).

However, the dsRNA production cost is still remarkably higher, although dsRNA production costs were lowered to 2 USD per gram in 2017 from 12,500 USD per gram back in 2008 (Zotti et al., 2018). The current dsRNA production capacity may be increased dramatically soon to produce vaccines against the COVID-19 (SARS-CoV2) pandemic to the global community. Furthermore, the same vaccine production platforms could be converted to produce large-scale RNAi-based pesticides, enabling dsRNA-based pesticides to be much cheaper (Taning C.N.T. et al., 2020).

It is a well-known phenomenon that the symbiont, blue-stain fungi pave the way for the successful colonization of coniferous bark beetles by acting as a source of bark beetles semiochemicals (Kandasamy et al., 2016), depletes spruce defense chemicals (Lahr and Krokene, 2013), and provide nutrient supplements (Bentz and Six, 2006; Six and Wingfield, 2011; Davis et al., 2019; Six, 2020; Six and Elser, 2020). Likewise, fungi also benefit from bark beetles getting inoculated into the phloem as they cannot penetrate bark alone (Franceschi et al., 2000). Thus, it concludes that the conifer bark beetles and their symbiont collectively causing to extensive Norway spruce forest mortality. For that reason, the researchers should work simultaneously on controlling symbionts and bark beetles by using the RNAi tool. Most interestingly, in our current research, we have seen that the RNAi is functional and highly efficient against the European spruce bark beetle, *Ips typographus*, in laboratory conditions (unpublished results).

Foliar application of RNAi-based pesticides may not be applicable for controlling the wood-boring insect pests due to the size of the trees and the presence of thick outer bark. In comparison, the delivery of dsRNA pesticides through the host such as trunk injection, soil drench, symbiotic microorganisms of plant and target pest, and viruses may promise the long-lasting protection of trees from insect pests and pathogens.

Restricting the off-target and non-target effects would be challenging with RNAi-based FPPs. However, species-specific and target-specific RNAi targets have to be identified for the effectiveness of this technology (Christiaens et al., 2018). A bioinformatics pipeline can be helpful here in finding HDRs in the target pest. However, the genome sequences of forest pests will be prerequisites for such strategies. Hence, research endeavors toward more forest insect genomes and tissue-specific transcriptomes are necessary for the future to obtain superior species-specific targets for dsRNA applications.

Lastly, the resistance against dsRNA-based FPPs would be a critical barrier for the deployment of this strategy. For example, Khajuria et al. (2018) reported that WCR got resistant upon exposure of several generations to *DvSnf7* dsRNA. Insect pests could evolve resistance in different possible ways. For instance, mutations of target and RNAi core machinery genes decreased dsRNA uptake and increased dsRNA degradation (Zhu and Palli, 2020). However, changes in the target gene selection could help delay the resistance, and delivery of dsRNA through nanoparticles and liposomes could improve the efficacy, stability, and dsRNA uptake mechanism by gut epithelial cells (Wytinck et al., 2020). The careful optimization of target gene selection, dsRNA design, synthesis and delivery, nanocarriers or symbiotic microbes, or virus-induced gene silencing can be highly effective for suppressing and causing mortality in forest insect pest species RNAi. However, field tests, environmental safety, and non-target effects are lacking for these efforts, and optimization is necessary for producing various RNAi-based FPPs. In addition, researchers must take the initiative to create a perception of RNAi-based FPPs to state forest agencies, forest owners, and general foresters to facilitate its deployment for long-term forest protection. Nevertheless, RNAi-based FPPs in conjugation with existing forest pest management practices (i.e., silvicultural, biological) can aid a multi-faceted management approach that keeps the tree-killing forest pest populations in the endemic stage while conserving the beneficial species.

## Author Contributions

AR conceptualized the manuscript structure. MJ, KM, and AR wrote the first draft and did the revision. GS commented on the initial draft and revised the manuscript. All the authors approved the final version of the manuscript.

## Conflict of Interest

The authors declare that the research was conducted in the absence of any commercial or financial relationships that could be construed as a potential conflict of interest.

## Publisher’s Note

All claims expressed in this article are solely those of the authors and do not necessarily represent those of their affiliated organizations, or those of the publisher, the editors and the reviewers. Any product that may be evaluated in this article, or claim that may be made by its manufacturer, is not guaranteed or endorsed by the publisher.
